# Clinical benefit without sweat chloride response after ETI therapy in an adult with cystic fibrosis bearing the L467F;F508del complex *CFTR* allele: a case report and the role of AI-assisted chest CT in longitudinal monitoring

**DOI:** 10.3389/fmed.2026.1930172

**Published:** 2026-08-28

**Authors:** Marcela Kreslova, Daan Caudri, Isabelle Sermet-Gaudelus, Aurelie Hatton, Punitkumar Makani, Vaclav Koucky, Malgorzata Libik, Hana Hrdinova, Libor Fila, Nela Stastna, Eva Furstova, Radka Bittenglova, Jan Baxa, Josef Sykora, Milan Macek

**Affiliations:** 1 Department of Paediatrics, University Hospital, Faculty of Medicine in Pilsen, Charles University, Pilsen, Czechia; 2 Department of Paediatrics, Division of Respiratory Medicine and Allergy, Erasmus MC–Sophia Children’s Hospital, University Medical Centre Rotterdam, Rotterdam, Netherlands; 3 Department of Radiology and Nuclear Medicine, Erasmus MC–Sophia Children’s Hospital, University Medical Centre Rotterdam, Rotterdam, Netherlands; 4 Institut Necker Enfants Malades, INSERM, Université Paris Cité, Paris, France; 5 Department of Paediatrics, Second Faculty of Medicine, Charles University, Motol and Homolka University Hospital, Prague, Czechia; 6 Department of Biology and Medical Genetics, Second Faculty of Medicine, Charles University, Motol and Homolka University Hospital, Prague, Czechia; 7 Department of Imaging Methods, Second Faculty of Medicine, Charles University, Motol and Homolka University Hospital, Prague, Czechia; 8 Department of Pneumology, Second Faculty of Medicine, Charles University, Motol and Homolka University Hospital, Prague, Czechia; 9 Department of Paediatrics, First Faculty of Medicine, Charles University, Thomayer University Hospital, Prague, Czechia; 10 Department of Pneumology, University Hospital, Faculty of Medicine in Pilsen, Charles University, Pilsen, Czechia; 11 Department of Imaging Methods, NH Hospital, AKESA Holding, Horovice, Czechia

**Keywords:** AI-assisted CT, case report, complex *CFTR* allele, cystic fibrosis, ETI (elexacaftor/tezacaftor/ivacaftor)

## Abstract

**Background:**

Elexacaftor/tezacaftor/ivacaftor (ETI) is the standard of care for most people with cystic fibrosis (CF) who carry at least one F508del allele. However, predicting responses to CFTR modulators in rare or complex *CFTR* genotypes is challenging. Sweat chloride concentration (SCC) and patient-derived functional assays assessing CFTR-mediated chloride transport are commonly used to classify modulator responsiveness. Carriers of the p.Leu467Phe;p.Phe508del (henceforward legacy nomenclature: L467F;F508del) complex *CFTR* allele have been considered unlikely to respond to ETI, based on short-term clinical, biomarker and *ex vivo* evidence. We report a discordant long-term response in an adult carrying this complex allele, with clinically meaningful benefit despite persistently negative CFTR chloride transport biomarkers.

**Case presentation:**

We describe an 8-year longitudinal pre- and post-treatment assessment of an adult woman with CF, adherent to therapy, with the L467F;F508del complex allele compounded with the non-responsive c.489 + 1G > T (621 + 1G > T) variant in *trans*. She has pancreatic insufficiency, progressive lung disease, and recurrent pulmonary exacerbations. After ETI commencement (at 40.5 years; July 2021) SCC remained unchanged at ∼100 mmol/L. Intestinal organoid and primary nasal epithelial cell assays showed no measurable ETI-related CFTR-mediated chloride transport rescue. Nevertheless, BMI increased from 22.0 to 26.8 kg/m^2^, annual exacerbations decreased from 5 to 3, and ppFEV₁ improved from 51% to 55–59%. Serial chest CT showed a shift from pre-ETI progression to post-ETI stabilisation, with reductions in air trapping and the extent of bronchiectasis on manual Brody II scoring and LungQ/PRAGMA-AI quantification. Exploratory *in vitro* exposure of nasal epithelial cells to vanzacaftor/tezacaftor/ivacaftor yielded ∼5% CFTR-mediated rescue, contrasting with the negative ETI response.

**Conclusion:**

Persistent CFTR biomarker negativity may not fully exclude clinically meaningful ETI-associated benefit in rare or complex *CFTR* genotypes. The pulmonary and extrapulmonary improvements observed in this case support multidimensional response assessment beyond single-biomarker endpoints. These hypothesis-generating observations support individualised long-term monitoring and comprehensive interpretation of CFTR biomarkers in the clinical context. AI-assisted chest CT may provide complementary real-world evidence. An exploratory *in vitro* rescue study with vanzacaftor/tezacaftor supports the evaluation of emerging CFTR modulators. It suggests that similar studies should be conducted in other cases involving complex *CFTR* alleles.

## 1 Introduction

People with cystic fibrosis (pwCF) who carry at least one p.Phe508del (legacy nomenclature: F508del) variant in CF transmembrane conductance regulator (*CFTR*) benefit from elexacaftor/tezacaftor/ivacaftor (ETI) CFTR modulator therapy (CFTRm). In Czechia, more than 81% of ETI-eligible pwCF, mostly with at least one F508del, receive ETI, which rapidly lowers sweat chloride concentration (SCC) and improves lung function, nutritional status, and quality of life (QoL) (1, 2). However, several of our ETI-eligible pwCF show non-responsive post-treatment CFTR chloride transport rescue biomarker outcomes and atypical clinical trajectories, suggesting that clinically relevant effects are not fully captured by chloride transport-based *in vitro* analyses alone.

In the absence of *CFTR* sequencing, current diagnostic approaches that target a specific panel of “candidate variants” miss complex alleles (c*CFTR*a), defined by multiple variants in *cis* (i.e., within the same gene); c*CFTR*a may modify the ETI biomarker response (3) in seemingly eligible variants, such as F508del, when the corresponding *CFTR* allele in *trans* is non-responsive. *CFTR* sequencing and parental variant phasing address this limitation, but these approaches are not yet part of routine diagnostic practice (3).

Theratyping and/or clinical observational studies (4) have broadened the range of rare *CFTR* variants predicted to respond to ETI (5), and the expected full clinical benefit is usually observed within weeks post administration (6). However, the magnitude of response may depend on age at treatment initiation, pre-existing structural lung damage, and the *CFTR* genotype, including the presence of c*CFTR*a. CFTRm-related clinical effects may also extend beyond measurable rescue of CFTR-mediated chloride transport, including reduced airway infections and inflammation (7). In addition, improvements in QoL and nutritional status may not be directly captured by biomarkers that assess rescue of CFTR-mediated chloride transport (8).

## 2 Case presentation

The adult woman with CF (awCF) was clinically diagnosed in 1982, at 2 years of age, and consistently demonstrated excellent adherence to CF care throughout follow-up. She had the classical CF phenotype, including pancreatic insufficiency from adolescence (faecal elastase I, 15 µg/g), progressive CF lung disease with bronchiectasis, mucus plugging, bronchial wall thickening and air trapping, frequent acute pulmonary exacerbations (aPEX), and early chronic *Pseudomonas aeruginosa* airway infection.

Microbiological surveillance was performed approximately six times per year using adequate-quality, spontaneously expectorated sputum samples. Chronic airway infection with *Staphylococcus aureus* had been documented since age 7 years, followed by chronic *P. aeruginosa* airway infection from age 9 years. *Stenotrophomonas maltophilia* and *Haemophilus haemolyticus* were also detected during follow-up. There was no evidence of allergic bronchopulmonary aspergillosis, nontuberculous mycobacterial pulmonary disease, or any clinically significant fungal pulmonary infection. Because adequate sputum samples were consistently available, bronchoscopy with bronchoalveolar lavage sampling was not performed. The patient received long-term inhaled antipseudomonal antibiotic therapy, predominantly colistin. Serial sputum cultures confirmed persistent chronic airway infection with *S. aureus* and *P. aeruginosa*, with no marked change in the overall microbiological pattern after ETI initiation.

Her concomitant chronic CF therapy also comprised once-daily inhaled dornase alfa, nebulised hypertonic saline once or twice daily, pancreatic enzyme replacement therapy, and regular respiratory physiotherapy incorporating airway clearance techniques. A Simeox device was incorporated into her airway clearance regimen in 2018.

**Figure 1:**
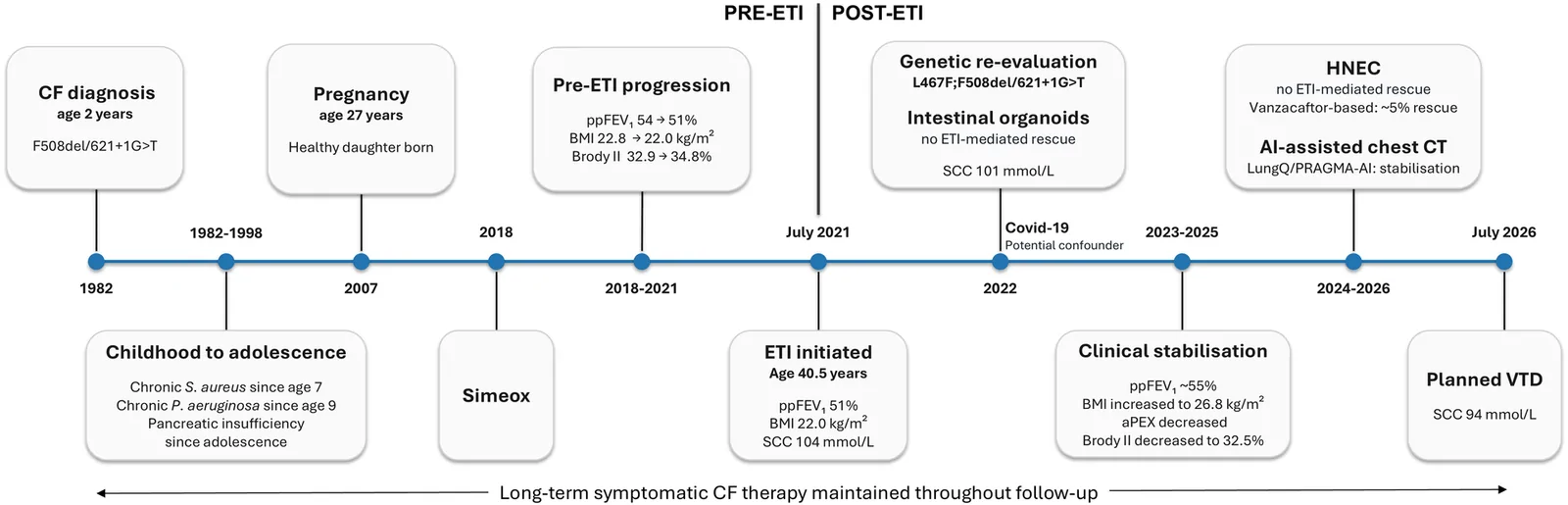
Integrated timeline of the clinical course and multimodal assessment of ETI response in an awCF carrying the L467F;F508del/c.489 + 1G > T c*CFTR*a. The timeline summarises the clinical course, long-term CF therapy, and multimodal assessment of ETI response. The blue horizontal axis represents chronological follow-up, and the vertical line marks ETI initiation in July 2021, at age 40.5 years, separating the pre- and post-ETI periods. The initially reported genotype was F508del/c.489 + 1G > T. Extended *CFTR* sequencing and phasing performed in 2022 identified the L467F;F508del c*CFTR*a in *cis*, with c.489 + 1G > T (legacy nomenclature: 621 + 1G > T) in *trans*. Intestinal organoid and HNEC testing showed no ETI-mediated rescue, whereas exposure of HNECs to vanzacaftor/tezacaftor/ivacaftor resulted in ∼5% CFTR-mediated rescue. The impact of COVID-19 around 2022 is indicated as a potential confounder. Long-term symptomatic CF therapy was maintained throughout follow-up. The planned switch to VTD is shown following the 5-year post-ETI assessment; VTD had not yet been initiated at the time of manuscript preparation. Chronological intervals are not drawn to scale. Detailed longitudinal data are provided in Table 1. AI, artificial intelligence; aPEX, acute pulmonary exacerbations; awCF, adult woman with cystic fibrosis; BMI, body mass index; CF, cystic fibrosis; CFTR, cystic fibrosis transmembrane conductance regulator; c*CFTR*a, complex *CFTR* allele; COVID-19, coronavirus disease 2019; CT, computed tomography; ETI, elexacaftor/tezacaftor/ivacaftor; HNEC, primary human nasal epithelial cells; LungQ, automated chest CT analysis platform; ppFEV₁, percent predicted forced expiratory volume in 1 second; PRAGMA-AI, automated artificial intelligence-assisted implementation of PRAGMA-CF; SCC, sweat chloride concentration; VTD, vanzacaftor/tezacaftor/deutivacaftor.

She has a university education, is married, and remained in full-time employment as a physician. She was a lifelong non-smoker, reported no substance misuse, and had no relevant non-CF comorbidities. She had no history of CF-related diabetes, CF-associated liver disease, or nasal polyposis. There was no known family history of CF or any another relevant inherited disorder. Her reproductive history comprised one uncomplicated pregnancy, resulting in the caesarean delivery of a healthy daughter at the age 27. She had strong family support. An integrated timeline of the principal clinical, diagnostic, and therapeutic milestones is provided in Figure 1.

**Table 1:** Clinical, imaging, laboratory and spirometry characteristics in the Czech awCF with c*CFTR*a L467F;F508del.

Pre- and post-ETI period (months)	−36	−24	−12	0	+6	+12	+18	+24	+36	+48
Years	2018	2019	2020	2021	2021	2022	2023	2023	2024	2025
Brody CT II score (%)										
Bronchiectasis	37.5	37.5	37.5	37.5	-	37.5	-	37.5	37.5	37.5
Airway wall thickening	29.6	33.3	33.3	35.2	-	37.0	-	35.2	33.3	33.3
Mucus plugging	41.7	44.4	44.4	44.4	-	44.4	-	44.4	44.4	44.4
Parenchyma	5.6	5.6	7.4	9.3	-	13.0	-	9.3	7.4	7.4
Air trapping	70.4	70.4	64.8	64.8	-	70.4	-	70.4	59.3	51.9
Total score	32.9	34.2	34.0	34.8	-	36.6	-	35.4	33.3	32.5
Automated LungQ Analysis^a^										
LungQ-LungVolume										
Inspiratory lung volumes (mL)	6208	5441	6302	5834	-	6117	-	6089	5435	5685
LungQ-PRAGMA-AI										
Disease (%)	11.26	10.35	7.21	11.40	-	10.68	-	5.81	7.28	7.19
Bronchiectasis (%)	10.11	6.07	4.29	8.38	-	6.40	-	3.42	5.23	2.19
Bronchial wall thickening (%)	1.15	4.28	2.29	3.02	-	4.28	-	2.40	2.05	5.00
LungQ-BA ratios (G1-G6)										
B_out_/A	1.73	1.27	1.28	1.54	-	1.42	-	1.27	1.51	1.10
B_in_/A	1.17	0.79	0.78	0.96	-	0.88	-	0.78	0.95	0.67
B_wt_/A	0.26	0.23	0.22	0.28	-	0.26	-	0.23	0.26	0.21
B_wa_/B_oa_	0.50	0.59	0.56	0.58	-	0.58	-	0.58	0.56	0.59
LungQ-MP										
Mucus plugs	128	199	199	250	-	311	-	181	238	272
Mucus plugs Volumes (mL)	9.51	27.60	30.65	25.74	-	48.07	-	24.89	45.66	36.18
SCC (mmol/L)^c^	-	-	-	104	101	101	100	-	-	-
BMI (kg/m^2^)	22.8	21.8	22.2	22.0	24.9	26.1	25.7	26.4	26.8	26.8
FEV_1_ (Litres)	1.46	1.41	1.37	1.34	1.39	1.44	1.44	1.55	1.51	1.40
ppFEV_1_ (%)	54	52	52	51	53	55	55	59	59	55
ppFVC (%)	71	49	71	66	77	62	61	65	63	64
ppFEV_1_/FVC (%)	80	113	76	82	73	94	98	99	99	91
aPEX-iv (yearly)	1	1	1	1	-	1^b^	-	1	1	1
aPEX-po (yearly)	5	5	5	5	-	3	-	2 + 1^b^	3	3

Evolution of clinical, laboratory, pulmonary function, and structural parameters before and during ETI therapy. The data show that SCC levels remained close to baseline due to c*CFTR*a, in contrast to disease stabilisation achieved under ETI treatment. However, this was complicated by the adverse effects of a COVID-19 infection. While ETI therapy led to significant nutritional improvements, reflected by an increase in BMI, the corresponding improvement in lung function (FEV_1_) was modest. Structural lung changes, assessed by the Brody CT II score, regressed more slowly, with notable reductions in air trapping and parenchymal abnormalities, alongside a slight decrease in airway wall thickening. Notably, there was no progression of bronchiectasis or mucus plugging. LungQ metrics reflect improved ventilation distribution and stability of structural changes.

aPEX-iv, acute pulmonary exacerbations with intravenous antibiotics; aPEX-po, acute pulmonary exacerbations with oral antibiotics; BA-ratio, Bronchial-Artery Ratio; B_out_/A ratio, Bronchial outer diameter artery ratio; B_in_/A ratio, Bronchial inner diameter artery ratio; B_wa_/B_oa_, Bronchial wall area to bronchial outer area ratio; BMI, body mass index; c*CFTR*a, complex CFTR allele; CT, computed tomography; ETI, Elexacaftor/Tezacaftor/Ivacaftor; FEV_1_, forced expiratory volume in 1 second; LungQ-LVA, Total lung volume analysis; LungQ-MP, Mucus Plug Quantification; ppFEV_1_, percent predicted forced expiratory volume in 1 second; PRAGMA-AI, LungQ automated version of the Perth-Rotterdam Annotated Grid Morphometric Analysis for CF; SCC, sweat chloride concentration.

a AI-based CT Analysis using the LungQ system developed by Thirona.

b COVID-19-related complication.

c SCC remained elevated at 94 mmol/L at +60 months (5 years post-ETI).

SCC was examined using the Macroduct Advanced Sweat Collection system (Elitech, USA). The C*FTR* gene was sequenced using an amplicon massively parallel sequencing assay (Devyser.com; Sweden) on the MiSeq platform (Illumina.com; USA), and the c*CFTR*a linkage phase was confirmed. ETI effects were assessed annually using aPEX frequency and duration, spirometry, body mass index (BMI), and chest computed tomography (CT). Structural lung disease was evaluated using manual Brody II scoring (9) and blinded automated AI-assisted LungQ analysis (Thirona; the Netherlands). Automated metrics included bronchus-artery ratios reflecting airway dilatation and wall thickening (10), a PRAGMA-AI percentage of structurally abnormal lung tissue, and LungQ mucus-plug quantification, including number and volume (11). Manual and automated CT assessments were compared descriptively across the pre- and post-ETI periods, using ETI initiation as baseline. Rescue of CFTR-mediated chloride transport was assessed in two independent *in vitro* patient-derived models: intestinal organoids (IOs) and nasal primary epithelial cultures (5, 12).

ETI was initiated at the standard-recommended adult dose in July 2021 at age 40.5 years. The regimen comprised two fixed-dose combination tablets, each containing elexacaftor 100 mg, tezacaftor 50 mg, and ivacaftor 75 mg, taken in the morning, followed by one ivacaftor 150-mg tablet in the evening, approximately 12 h apart. Both doses were taken with fat-containing food and pancreatic enzyme replacement therapy. ETI was continued at the full dose without reduction or interruption throughout follow-up and was well tolerated, with no reported ETI-related adverse events, including sleep disturbances or psychological symptoms. The established chronic symptomatic CF treatment regimen was maintained after ETI initiation, with no new chronic therapy introduced and none discontinued or intensified.

Adherence to ETI and background therapy was assessed at each outpatient visit through targeted patient interviews, medication reconciliation including patient-reported quantities of medication remaining at home, and review of pharmacy dispensing records confirming regular ETI collection. All concomitant medications were reviewed, and no clinically relevant drug–drug interactions were identified; specifically, she was not taking any CYP3A-inducing agents expected to reduce ETI exposure.

**Figure 2:**
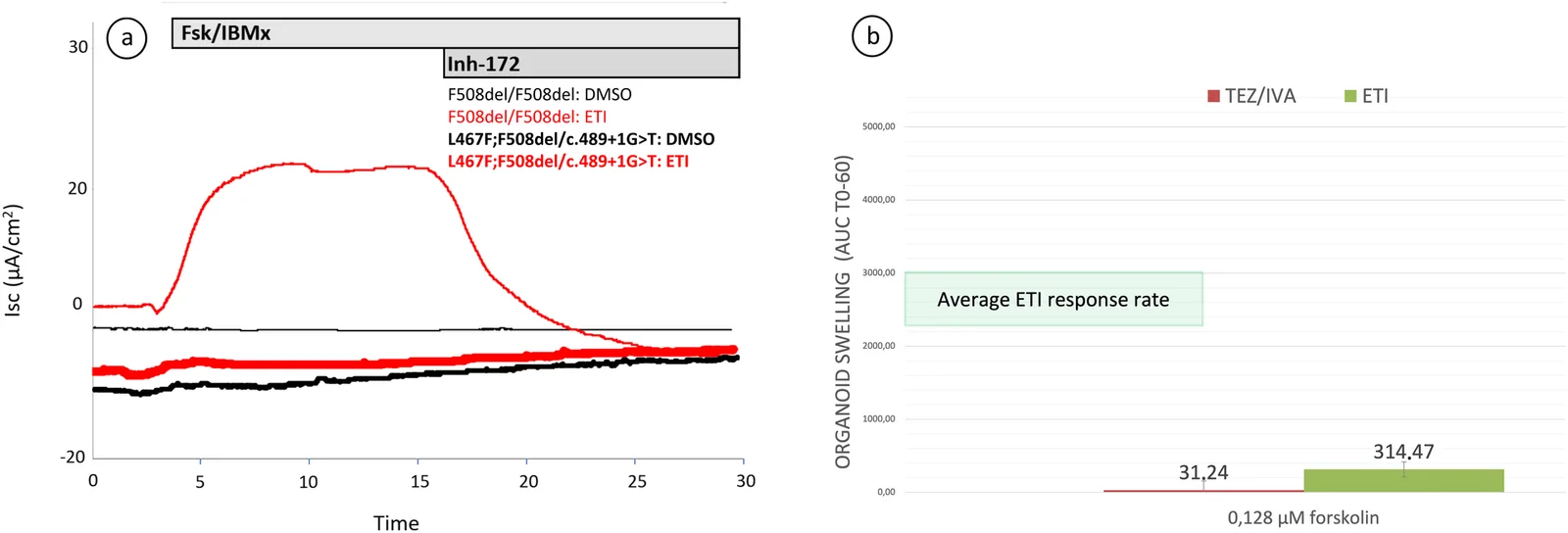
*In vitro* results using ETI in primary nasal epithelial cells of an awCF carrying the L467F;F508del/c.489 + 1G > T c*CFTR*a. ⓐ Ussing's representative tracing in primary nasal epithelial cells from the patient incubated for 48 hours with DMSO (vehicle) (black) or elexacaftor (3 µM)/tezacaftor (3 µM)/ivacaftor (100 nM), (ETI) (red) as compared to cells from a patient F508del homozygous. Activation of the cAMP-dependent Cl- secretion by Forskoline (10 µM)/IBMX (100 µM) and selective inhibition of the CFTR-dependent Cl- secretion by In-172 (5 µM). Restoration of the CFTR-dependent Cl- secretion in F508del homozygous cells by ETI (thin line) as compared to the L467F;F508del/c.489 + 1G > T patient cells, where no response is observed. ⓑ FIS assay performed on patient-derived IOs showed almost no response to tezacaftor/ivacaftor (red) and practically no response to ETI (green). Graphs show the average AUC after an 11-hour experiment and the standard deviation across 3 replications, using a reference concentration of 0.128 M of forskolin (commonly used across laboratories and publications). For reference, we show a green box representing the range of average *in vitro* ETI responses in a cohort of pwCF harbouring at least one copy of F508del. awCF, adult with cystic fibrosis; AUC, area under the curve; c*CFTR*a, complex *CFTR* allele; CF, cystic fibrosis; CFTR, cystic fibrosis transmembrane conductance regulator; DMSO, dimethyl sulfoxide; ETI, elexacaftor/tezacaftor/ivacaftor; FIS, forskolin-induced swelling; IBMX, 3-isobutyl-1-methylxanthine; IOs, intestinal organoids.

**Figure 3:**
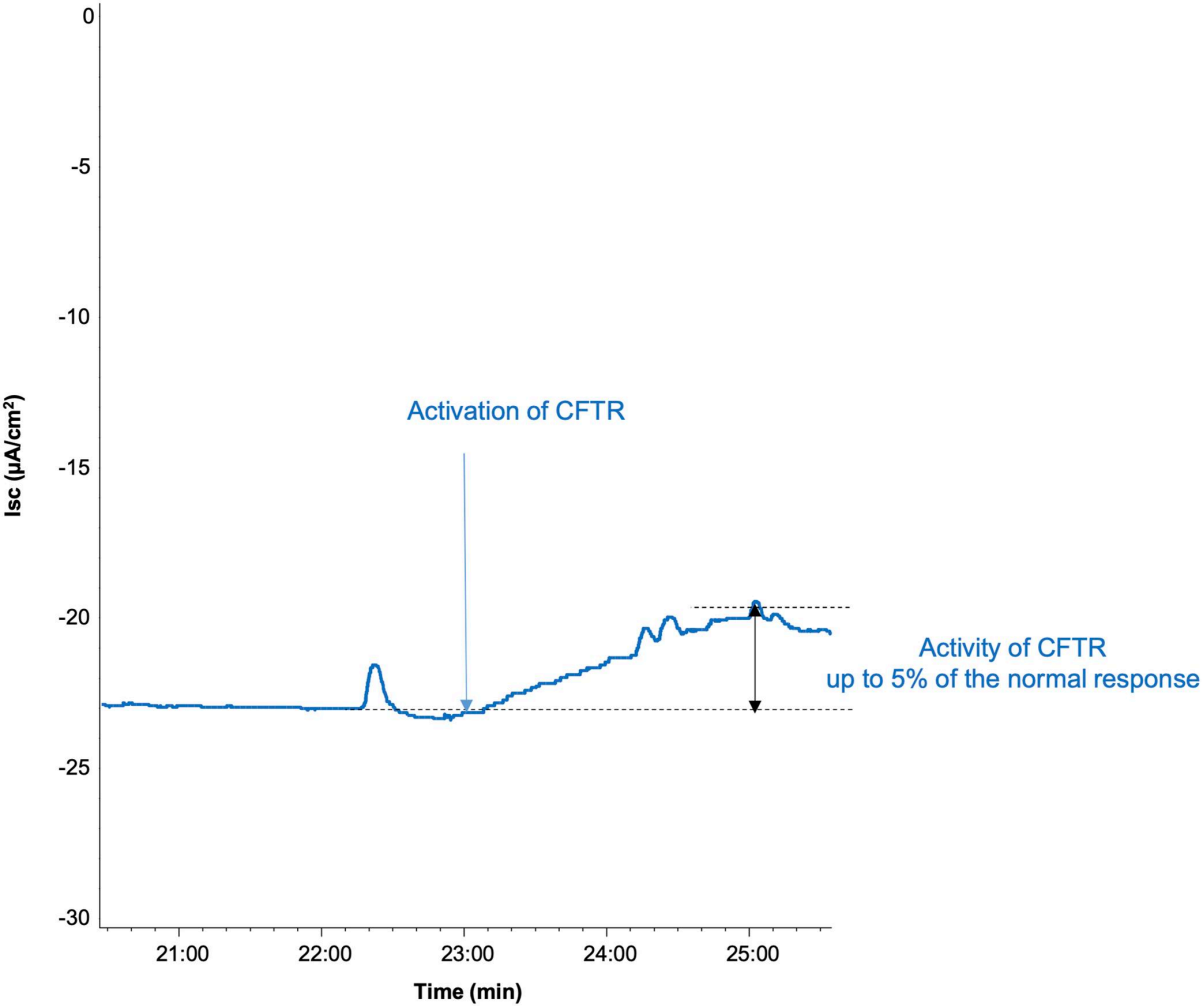
*In vitro* results in primary nasal epithelial cells obtained from awCF carrying the L467F;F508del/c.489 + 1G > T c*CFTR*a, following tezacaftor/vanzacaftor and ivacaftor exposure. A low but detectable CFTR-mediated response of ∼5% was observed after vanzacaftor/tezacaftor followed by ivacaftor exposure. Ussing's representative tracing in primary nasal epithelial cells from the awCF with the c*CFTR*a were incubated for 48 hours with VX-661/tezacaftor (10 µM) and VX-121/vanzacaftor (1 µM), followed by acute VX-770/ivacaftor (0.1 µM) exposure. cAMP-dependent Cl⁻ secretion was stimulated by forskolin (10 µM), showing CFTR activation reaching up to 5% of the normal response. awCF, adult with cystic fibrosis; cAMP, cyclic adenosine monophosphate; c*CFTR*a, complex *CFTR* allele; CFTR, cystic fibrosis transmembrane conductance regulator; Cl^−^, chloride ion.

However, her mean SCC remained close to baseline throughout follow-up (104 mmol/L at baseline vs. 101 mmol/L after 6 months and 94 mmol/L after 5 years of ETI; see Table 1). Subsequent *CFTR* gene sequencing detected the L467F;F508del c*CFTR*a in *cis* and an “ETI-nonresponsive” variant in *trans* (c.489 + 1G > T, legacy nomenclature: 621 + 1G > T). Repeated IOs and nasal epithelial cultures exposed to ETI demonstrated no restoration of CFTR-mediated chloride transport, with an absent forskolin response and a subsequent response to the CFTR-specific inhibitor Inh-172 (Figure 2). Nonetheless, recent *in vitro* vanzacaftor/tezacaftor/ivacaftor testing in her nasal epithelial cultures showed ∼5% CFTR-mediated rescue (Figure 3).

Despite the absence of measurable CFTR-dependent chloride transport after ETI administration, clinically apparent improvement was observed over a 4-year post-ETI period (Table 1). BMI increased from 22.0 to 26.8 kg/m^2^, indicating improved nutritional status. The annual number of aPEX treated with oral antibiotics decreased from 5 to 3 with shorter treatment durations, while the need for intravenous antibiotics remained stable at no more than once yearly. Subjectively, nasal obstruction improved, while ppFEV_1_ increased from 51% at baseline to 59% at 24 and 36 months, and remained above baseline at 48 months (55%). Absolute FEV_1_ increased from 1.34 L (L) at baseline to 1.55 L at 24 months and was 1.40 L at 48 months. Although forced vital capacity (FVC) oscillated, particularly during the COVID-19 pandemic, functional decline did not progress, in contrast to the gradual progression observed on the pre-ETI CT. Her structural lung disease stabilised after commencing ETI (Table 1).

**Figure 4:**
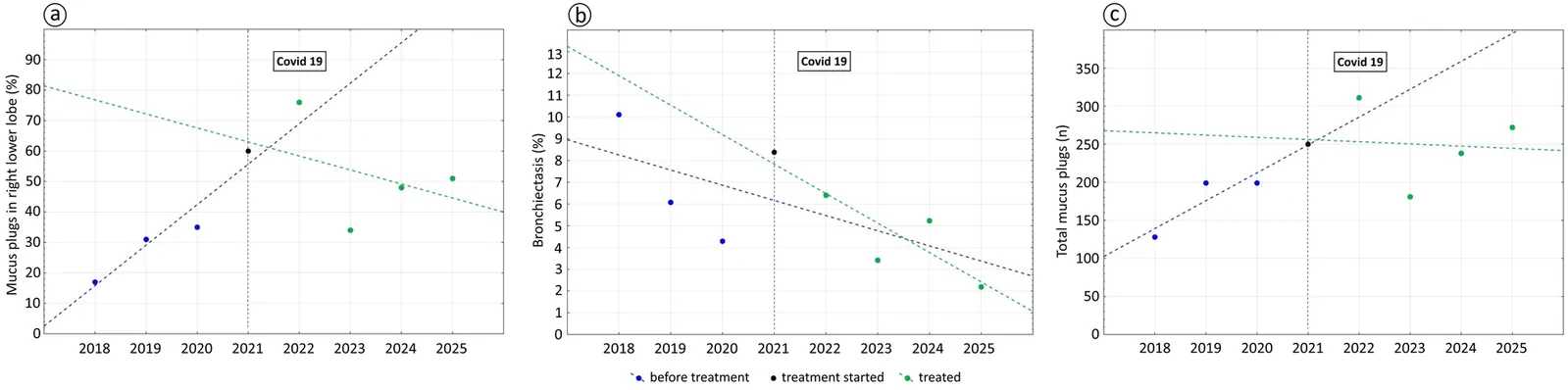
Representative longitudinal regression patterns of CT-derived structural lung disease parameters before and after ETI initiation in an awCF with the L467F;F508del c*CFTR*a (PRAGMA-AI). ⓐ Representative graph showing reversal of the pre-treatment worsening trend after ETI initiation, reflected by a decrease in mucus plug burden in the right lower lobe (%). ⓑ Representative graph showing a steeper post-treatment decline in bronchiectasis (%) after ETI initiation. ⓒ Representative graph showing attenuation of the pre-treatment progression trend after ETI initiation, reflected by stabilisation of total mucus plugs **(n)**. Blue points and the blue dashed regression line represent pre-ETI values; green points and the green dashed regression line represent post-ETI values. The black dot and the vertical dashed line indicate the time point of ETI initiation. Data points corresponding to 2022 and 2023 (+12 and +24 months post-ETI initiation, respectively) coincided with COVID-19-related perturbations, representing a potential confounder. awCF, adult with cystic fibrosis; c*CFTR*a, complex *CFTR* allele; CT, computed tomography; ETI, elexacaftor/tezacaftor/ivacaftor.

Both visual and AI-assisted CT analyses demonstrated a change in trajectory from pre-treatment progression to post-treatment stabilisation (Figure 4). Manual Brody II scoring confirmed pre-ETI progression followed by stabilisation after ETI initiation. Airway wall thickening decreased slightly from 35.2% to 33.3%, whereas air trapping decreased more markedly from 64.8% to 51.9%, suggesting reduced small-airway disease. Bronchiectasis and mucus plugging showed no visible progression. Serial visual review also indicated reduced expiratory tracheal flattening and greater parenchymal homogeneity, although these features were not quantified.

From 2022 onward, PRAGMA-AI showed a decrease in total CF lung disease from 11.4% to 7.19% during ETI therapy. LungQ-BA metrics indicated stabilisation of bronchial widening and wall thickening, with improvement in selected parameters, including a decrease in PRAGMA-AI bronchiectasis from 8.38% to 2.19% and in Bout/A from 1.54 to 1.10. Due to the single-case design, statistical analysis was not performed.

## 3 Discussion

We report discordance between current biomarker readouts assessing rescue of CFTR-mediated chloride transport and the objectively stabilised CF lung disease and overall improved clinical trajectory in a longitudinally monitored adult woman with CF receiving ETI and carrying the “revertant” complex *CFTR* allele (3) and an “ETI-non-responsive” *CFTR* variant in *trans*.

Thus, in patients in whom SCC remain at baseline after ETI administration and who were not sequenced, the presence of c*CFTR*a should be examined. The L467F;F508del c*CFTR*a attenuates response to ETI and has previously been associated with the classification of carriers as “non-responders” based on short-term biomarker and clinical grounds (3). Our case does not contradict the exclude previously documented impairment of CFTR-mediated chloride transport but suggests that persistent biomarker negativity does not preclude delayed, potentially tissue-dependent clinical benefit from ETI; responsiveness to CFTRm should therefore be interpreted multimodally over the long term (13). Recent real-world data from adults with CF, including pwCF ineligible for pivotal clinical trials, have also highlighted the broader intra- and extrapulmonary impact of ETI, supporting the need for multidimensional response assessment beyond single-biomarker endpoints (14, 15).

Our findings do not challenge the overall predictive value of SCC or theratyping but suggest that exceptions may occur. Although theratyping and SCC most often align with clinical outcomes, these analyses may not capture all clinically relevant effects in the airways (5) or reliably predict individual clinical outcomes (12). Indeed, CFTR is much more than a "simple" chloride channel (16). In this regard, Pranke et al. underscore knowledge gaps when *ex vivo* biomarkers and clinical outcomes diverge (16). N1303K-related data show respiratory improvement despite minimal SCC reduction, supporting tissue-dependent rescue following ETI therapy, although caution is warranted in drawing analogies with L467F;F508del.

The patient started ETI therapy in adulthood, after years of progressive CF lung disease, when irreversible airway remodelling likely occurred. In adults with advanced CF, established structural lung disease is unlikely to reverse substantially; therefore, lung disease stabilisation and a decrease in aPEX represent clinically relevant outcome measures (11, 17).

In this awCF, CT improvement was modest, but the pre-treatment progression trajectory markedly improved after ETI initiation. Automated CT analysis added sensitivity beyond Brody scoring, with PRAGMA-AI and LungQ-BA indicating a more favourable trajectory, supporting AI-assisted imaging as a complementary tool when CFTR transport-based biomarker readouts are inconclusive or discordant.

An alternative interpretation is that stabilisation occurred for reasons unrelated to ETI. However, her full adherence, the absence of changes in long-term multidisciplinary care, including nutritional management and chronic symptomatic CF therapy, and the lack of any marked shift in sputum microbiology during follow-up, together with the combined BMI gain, reduced aPEX burden, sustained ppFEV_1_ above baseline, and stabilised structural lung disease, argue against this notion.

### 3.1 Strengths and limitations

The principal strength of this report is the depth of longitudinal clinical, imaging and laboratory phenotyping in a rare and clinically challenging *CFTR* genotype. The case integrates pre- and post-ETI clinical follow-up, repeated SCC measurements, two independent patient-derived functional models, manual Brody II CT scoring, and blinded automated LungQ/PRAGMA-AI quantification. This multidimensional design is particularly informative for complex *CFTR* alleles, where a single readout may underrepresent the clinical phenotype. In our patient, a change from pre-ETI structural progression to post-ETI stabilisation was documented, despite persistently unchanged SCC and the absence of ETI-related CFTR-mediated chloride transport rescue *ex vivo*.

The relevant literature provides both contrasting and supportive contexts. Previous studies of L467F;F508del showed that this complex allele hampers ETI-mediated pharmacological rescue and supported classifying affected carriers as “non-responders” on short-term biomarker and clinical grounds (3, 13). Our SCC, intestinal organoid and nasal epithelial data are fully consistent with impaired chloride-transport rescue. However, recent reports indicate that *CFTR* variant responsiveness may differ across CFTR modulator combinations, including differential responses to vanzacaftor- versus elexacaftor-based treatment (18). Most directly, Capurro et al. described *ex vivo* prediction and exploratory clinical assessment of vanzacaftor-tezacaftor as an alternative therapeutic option for the ETI-resistant L467F;F508del allele (19). Therefore, lack of response to one modulator combination should not necessarily be extrapolated to all current or emerging CFTRm regimens. Our case contrasts with these reports by focusing on long-term, real-world ETI exposure with persistently negative chloride-transport biomarkers, and by adding serial AI-assisted chest CT as an objective structural outcome of disease stabilisation.

The limitations are inherent to an observational single-case report. Causality cannot be proven, and residual confounding by infection dynamics, microbiology, antibiotic exposure, physiotherapy, nutrition, adherence, regression to the mean, COVID-19-related perturbations, or other aspects of multidisciplinary care cannot be completely excluded. However, the awCF is fully adherent to therapy, and the impact of ETI is demonstrated beyond age 40, despite sparse clinical studies. Although patient-reported symptom changes were documented narratively during routine follow-up, no standardised longitudinal QoL instrument or other patient-reported outcome measure, such as the Cystic Fibrosis Questionnaire–Revised, was administered, precluding formal assessment of patient-perceived treatment benefit. The CT findings were interpreted descriptively, and no statistical inference was possible. Quantitative AI-assisted imaging may also be affected by acquisition parameters, differences in inspiratory and expiratory volumes, segmentation performance, and the still-evolving validation of automated CF CT metrics. Similarly, intestinal organoids and nasal epithelial cultures may not fully capture all airway compartments, inflammation, mucus properties, host modifiers, or extrapulmonary effects of CFTRm therapy. Finally, the adult age at treatment initiation and the presence of established bronchiectasis limit generalisability to children, to less advanced CF lung disease, or to other complex *CFTR* alleles.

The main takeaway is that persistent SCC negativity and the absence of short-term *ex vivo* chloride-transport rescue should prompt careful reassessment of the *CFTR* genotype by sequencing the entire gene, including phasing for complex alleles. Still, it should not automatically preclude continued ETI therapy when the broader longitudinal phenotype suggests a clear benefit. In rare or complex *CFTR* genotypes, response should be assessed as a patient-specific trajectory across symptoms, aPEX frequency, nutrition, spirometry, microbiology, CT morphology, and patient-derived functional data. AI-assisted CT cannot replace established biomarkers or clinical judgment. Still, in biomarker-discordant cases, it may strengthen real-world evidence by providing reproducible, regionally resolved quantification of structural lung disease over time. We consider this case unique because it combines an ETI-biomarker-negative L467F;F508del complex allele with objective long-term clinical stabilisation documented by AI-assisted serial chest CT, thereby supporting a time-extended, multimodal framework for assessing modulator benefit and prioritising emerging alternatives such as the vanzacaftor/tezacaftor/deutivacaftor combination.

## 4 Patient perspective

After starting ETI, I noticed my breathing felt freer and clearer and my nasal obstruction improved. My respiratory condition became more stable in daily life, and I needed antibiotics less often when my cough worsened. However, contracting COVID-19 took quite a toll on me. Initially, I did not understand why my sweat chloride levels had not decreased, especially since I felt a bit better. Detailed genetic testing finally provided an explanation, which made me joke, “ I suppose I always have to be exceptional in some way”. I am grateful that I remain active and can continue working full-time. I have not experienced any noticeable treatment-related adverse effects. I sleep well, my appetite has greatly improved (sometimes almost too much), and I have no mood swings, unless I count those associated with raising a teenage daughter. Although my sweat chloride concentrations remain high, I consider the overall stabilisation of my condition a real benefit. I am very happy that ETI is reimbursed in our country, and I remain hopeful that in the future, even more effective drugs will become available even for me, given my specific genotype. Above all, I am deeply grateful to my CF team and my family for their long-term support, and I am absolutely overjoyed to have a beautiful, healthy daughter!

## 5 Conclusions

Although limited by its single-case design, our report provides comprehensive long-term clinical, functional, AI-assisted lung imaging, and *in vitro* biomarker data for an adult with CF carrying the L467F;F508del complex *CFTR* allele. The case suggests that persistently elevated baseline SCC values should not automatically preclude continued ETI when other clinical indicators indicate benefit. Therapeutic decisions should therefore integrate the broader clinical context with individualised long-term monitoring of nutritional status and CF lung disease trajectory, while also considering emerging CFTRm options (18, 20). Guided by the theratyping concepts (5), exploratory *in vitro* testing of vanzacaftor/tezacaftor combinations in our case demonstrated a potentially clinically relevant 5% rescue of CFTR-mediated chloride transport. The observed *in vitro* response supports further evaluation of vanzacaftor/tezacaftor/deutivacaftor therapy in additional individuals carrying the L467F;F508del c*CFTR*a, as recently reported (19). Longitudinal AI-assisted lung imaging may provide complementary real-world evidence for assessing treatment response in such rare or complex *CFTR* genotypes, particularly when conventional CFTR biomarker readouts are inconclusive or discordant.

## Data Availability

The raw data supporting the conclusions of this article will be made available by the authors, without undue reservation.
